# Xanthatin synergizes with cisplatin to suppress homologous recombination through JAK2/STAT4/BARD1 axis in human NSCLC cells

**DOI:** 10.1111/jcmm.16271

**Published:** 2021-01-13

**Authors:** Jian Zhang, Sheng Yang, Hongmei Guan, Jueyu Zhou, Yuan Gao

**Affiliations:** ^1^ Department of Medical Genetics School of Basic Medical Sciences Southern Medical University Guangzhou China; ^2^ Department of Biochemistry and Molecular Biology School of Basic Medical Sciences Southern Medical University Guangzhou China; ^3^ The First School of Clinical Medicine Southern Medical University Guangzhou China; ^4^ Nanfang Hospital Southern Medical University Guangzhou China

**Keywords:** antitumor, BARD1, cisplatin, homologous recombination, NSCLC, Xanthatin

## Abstract

Xanthatin (Xa) is a bicyclic sesquiterpene lactone identified from the plant *Xanthium L*. with impressive antitumor activity, but the role of Xa in non‐small cell lung cancer (NSCLC) is not known. Here we found that Xa inhibits proliferation, migration, invasion and induces apoptosis in NSCLC cells. RNA sequencing and Gene set enrichment analysis revealed that Xa significantly activates p53 pathway and suppresses E2F targets, G2M checkpoint and MYC targets in A549 cells. Among these changed genes, the down‐regulated gene BARD1 triggered by Xa was identified as a candidate involved in Xa’s antitumor effect because of its vital role in homologous recombination (HR). Further studies demonstrated that Xa inhibits HR through the BARD1/BRCA1/RAD51 axis, which enhances cell sensitivity to cisplatin. Mechanistic studies showed that Xa inhibits BARD1 through the JAK2/STAT4 pathway. Our study revealed that Xa is a promising drug to treat NSCLC, especially in combination with conventional chemotherapy.

## 1 INTRODUCTION

Lung cancer is the most common cancer and the leading cause of cancer‐related death in the world.^1^ Lung cancers generally fall into 2 broad categories, of which NSCLC accounts for 83% (the other category is small cell lung cancer, SCLC).^2^ The current survival rate of lung cancer patients has been improved with the occurrence of new drugs including PD‐L1 monoclonal antibody (Pembrolizumab),^3^ EGFR and ALK tyrosine kinase inhibitors.^4^ However, most patients without genetic mutation have minor or no response to those kinds of immunotherapies. As a result, platinum‐based chemotherapies are still the main methods for advanced lung cancer patients,^5^, ^6^ however, the typical chemotherapeutic agents are limited by its significant toxicity. Development of novel therapeutic agents is of significant need to improve the clinical outcomes of lung cancer patient.

Cisplatin (Cis) is one of the first line chemotherapeutic agent for treating advanced NSCLC. However, the clinical response rate of patients to Cis‐based chemotherapy regimens is only 15% to 30%.^7^, ^8^ Although the drug resistant mechanism is complicated, the abnormal enhancement of DNA repair capacity in cancer cells is accounted, which undermines the therapeutic efficacy of DNA damage‐inducing drugs such as Cis, etoposide and camptothecin.^9^ The Cis sensitization in NSCLC cells is tightly correlated with the activation status of DNA repair pathways.^10^ In terms of mechanism, cells activate DNA damage response (DDR) to safeguard genomic integrity in response to DNA damage. The DDR consists of six major DNA repair pathways.^11^ Two of these pathways, non‐homologous end joining (NHEJ) and homologous recombination (HR), dominate the repair of double‐strand breaks (DSBs).^12^ NHEJ directly ligates the broken DNA ends, which is considered to be error‐prone.^13^ By contrast, HR is an error free pathway that uses the information stored in a sister chromatin or homologue to repair the damage.^11^ More importantly, HR is the most effective pathway activated in tumor cells to perform DSBs repair induced by Cis.^14^, ^15^ Hence, it is an effective strategy to overcome cancer drug resistance by targeting HR pathway.^16^, ^17^

Xa is a bicyclic sesquiterpene lactone and widely exists in the plant *Xanthium L*.^18^ Xa has a broad spectrum of biological activities. Recent studies indicated that Xa has significant antitumor effects on a number of malignancies including lung cancer,^19^ hepatocellular carcinoma,^20^ colon cancer^21^ and glioma.^22^ Xa triggers Chk1‐mediated DNA damage response in lung cancer cells.^19^ Furthermore, Xa induces endoplasmic reticulum (ER) stress, leading to activation of apoptosis in hepatoma cells.^20^ Moreover, Xa activates ROS/XIAP signaling pathway to mediate G2/M cell cycle block and autophagy in colon cancer cells.^21^ Nevertheless, the mechanisms of Xa’s antitumor effects have not yet been defined.

In this study, we investigated whether Xa could increase the sensitivity to DNA damage and how Xa could regulate the DNA repair process in NSCLC. We found that Xa induces DNA damage, thus consequently leads to cell cycle arrest and apoptosis through the p53 signaling pathway. Moreover, our results clearly demonstrated that Xa has significant synergistic effects with Cis and powerful anticancer efficacy through suppressing HR via JAK2/STAT4/BARD1 axis. These findings support potential applications of Xa as an anticancer therapeutic modality that targets HR pathway in NSCLC.

## 2 MATERIALS AND METHODS

### 2.1 Cell lines

The NSCLC cell lines A549 cells and H1299 cells were obtained from the Cell Bank of Chinese Academy of Sciences (Shanghai, China). Cells were cultured in RPMI‐1640 medium containing 10% fetal bovine serum (FBS) and then placed in a humidified atmosphere containing 5% CO_2_ at 37°C.

### 2.2 Chemicals and reagents

Xa was purchased from Yuannuotiancheng (Chengdu, China) and was dissolved in dimethyl sulfoxide (DMSO) as a stock solution at 40 mmol/L. JAK2 inhibitor TG101348 (GlpBio) was dissolved in DMSO as a stock solution at 10 mmol/L. All solutions stored at −80°C, and diluted in RPMI‐1640 before using.

### 2.3 Cell transfection

The small interfering RNAs (siRNAs) targeting RAD51 (si‐RAD51: AAGGGAAUUAGUGAAGCCAAATT) and siRNAs for control (NC) were obtained from GenePharma (Shanghai, China). The full‐length BARD1 cDNA was cloned into the pEZ‐M07 vector (GeneCopoeia, China) to construct BARD1‐expressing plasmid pEZ‐M07. Lipofectamine^™^ 2000 transfection reagent (Invitrogen) was used to perform transfection.

### 2.4 Cell viability analysis

Equal number of cells were plated in 96‐well plates and incubated at 37°C for 24 hours. Subsequently, the cells were treated with Xa (2.5, 5, 10, 20 and 40 μmol/L) or vehicle control for 12, 24 and 48 hours. Finally, 10 μL CCK‐8 reagent (Dojindo Laboratory, Japan) was added to each well and incubated at 37°C for another 2 hours. OD value (450 nm) was detected by a microplate reader for calculating cell viability.

### 2.5 Cell wound scratch assay

Cells were plated in 6‐well plates at a suitable density and were incubated at 37°C, 5% CO_2_. When cell confluence was 90%, cells were treated with serum‐free medium overnight. Subsequently, 10 μL pipette tip was used to scratch the bottom of the six‐well plate. Cells were cultured in no‐serum medium with or without Xa. The width of the scarification worked as an indicator of migration distance and was photographed under a microscope at 0 and 48 hours.

### 2.6 Transwell assay

The transwell insert with 8 μm pore (Corning) were placed on 24‐well plates. Subsequently, matrigel matrix was used to coat upper chambers (30 μL/chamber) and dried in air at 37°C for 2 hours. Cells (control or pre‐treated with Xa) were added to the upper chamber in serum‐free medium, while medium with 20% fetal bovine serum was added in the lower chamber. The 24‐well plates with transwell insert were placed at 37°C with 5% CO_2_ for 12‐24 hours. Cells in the upper chambers were carefully removed by using cotton swabs. Cells that migrated through the membrane were stained with 0.5% crystal violet and photographed under a microscope. The number of cells were calculated and used as indicator of invasion ability.

### 2.7 Hoechst 33258 staining

Cells were plated in 96‐well plates at a suitable density and incubated at 37°C, 5% CO_2_ for 24 hours. Subsequently, cells were treated with DMSO (control) or Xa for another 24 hours. 4% paraformaldehyde was used to fix the cells, then 5 μg/mL Hoechst 33258 was added to each well for 15 minutes. Finally, the nuclei morphologies in different cells were stained with bright blue and observed under a fluorescence microscope.

### 2.8 Cell cycle and apoptosis analysis

For cell cycle analysis, the cells were harvested by EDTA‐free trypsin and collected into centrifuge tubes, fixed with iced 70% ethanol overnight after washing. Propidium iodide (PI) solution (Yeasen, China) was added to each centrifuge tube and incubated for 30 minutes in the dark at room temperature after washing off the fixative with PBS. Finally, cell cycle was analyzed by flow cytometry.

For cell apoptosis analysis, cells were harvested by EDTA‐free trypsin and collected into centrifuge tubes. Subsequently, Annexin V‐FITC/PI (Yeasen, China) was added to each centrifuge tube and incubated for 30 minutes at room temperature. Finally, cell apoptosis was analyzed by flow cytometry.

### 2.9 RNA sequencing (RNA‐seq) and data analysis

Total RNA was extracted from cells for RNA‐seq using TRIzol reagent (Invitrogen; Thermo Fisher Scientific, Inc.), from three replicates of Xa treatment A549 cells (exposed to 10 μmol/L) and three replicates of controls. The quality and integrity of the RNA samples were examined as previously described.^23^ RNA‐seq was performed at Illumina Hiseq 2500 platform that included quality control, library preparation, fragmentation and PCR enrichment of target RNA according to standardized procedures. 150 bp paired‐end raw reads were initially processed to obtain clean reads by removing adaptor sequences, low quality sequences, empty reads. After quality control, the clean reads were mapped to human genome (hg38) using TopHat.^24^ Genes expression level were quantitated by FPKM (Fragments per kilobase of exon per million reads mapped). Genes were considered to be expressed if they had an FPKM value greater than 1 in at least three or more samples. Differentially expressed genes (DEGs) between control and Xa treatment cells were identified by using Cuffdiff.^25^ An absolute fold change >2 and a FDR significance score <0.05 were used as thresholds to identify DEGs. Gene set enrichment analysis (GSEA) was performed using GSEA software (http://software.broadinstitute.org/gsea/) with default parameters.

### 2.10 Validation of DEGs with quantitative real‐time PCR (qRT‐PCR)

In order to validate the reliability of the RNA‐seq analyses, 12 candidate DEGs (*NTRK3, HMOX1, ALDH1A3, DUSP6, BARD1, ZMAT3, FAS, IGFBP3, CCNB3, CDKN1A, BBC3, SESN1*) were selected for qRT‐PCR tests both in A549 and H1299 cells. RNA expression levels were normalized to *GAPDH*. Each sample was analyzed in triplicate and the mean expression level was calculated. Primer sequences for these genes were shown in Table S1.

### 2.11 Western blot (WB) analysis

Radioimmuno‐precipitation assay (RIPA) lysis buffer was used to extract WB analysis total protein from in vitro cultured cells. Proteins were resolved by sodium dodecyl sulfate polyacrylamyde gel electrophoresis (SDS‐PAGE) and wet transferred to polyvinylidene difluoride (PVDF) membranes. The membranes with proteins were subsequently blocked in 5% BSA and then incubated with antibodies specific to FAS (13098‐1‐AP; Proteintech), ALDH1A3 (25167‐1‐AP; Proteintech), ZMAT3 (10504‐1‐AP; Proteintech), IGFBP3 (10189‐2‐AP; Proteintech), CCNB3(AB44527; Absci), BBC3 (55120‐1‐AP; Proteintech), SESN1 (21668‐1‐AP; Proteintech), NTRK3 (A14033; ABclonal), DUSP6 (A3171; ABclonal), HMOX1(10701‐1‐AP; Proteintech), CDKN1A(10355‐1‐AP; Proteintech), BARD1 (A1685; ABclonal), BRCA1 (A0212; ABclonal), RAD51 (14961‐1‐AP; Proteintech), γH2AX (AP0099; ABclonal), NTRK3 (A14033; ABclonal), JAK2 (A19629; ABclonal), Phospho‐JAK2‐Y1007/1008 (p‐JAK2; AP0531; ABclonal), STAT4 (A4523; ABclonal), Phospho‐STAT4‐Y693 (p‐STAT4; AP0137; ABclonal). Subsequently the membranes were incubated with secondary antibody conjugated with HRP and finally ECL (Millipore) was used to measure the protein bands semiquantitatively and normalized to the gray value of GAPDH.

### 2.12 Statistical analysis

All results were expressed as mean ± SD. and analyzed using Graphpad Prism7 software. All experiments were repeated at least three times. Student’s *t* test was used to determine the significance between two groups. One‐way analysis of variance with Tukey test was used for multiple comparisons. *P < 0.05, **P < 0.01 and ***P < 0.001 were considered statistically significant.

## 3 RESULTS

### 3.1 Xa suppresses cell proliferation, migration and invasion in NSCLC cells

**FIGURE 1:**
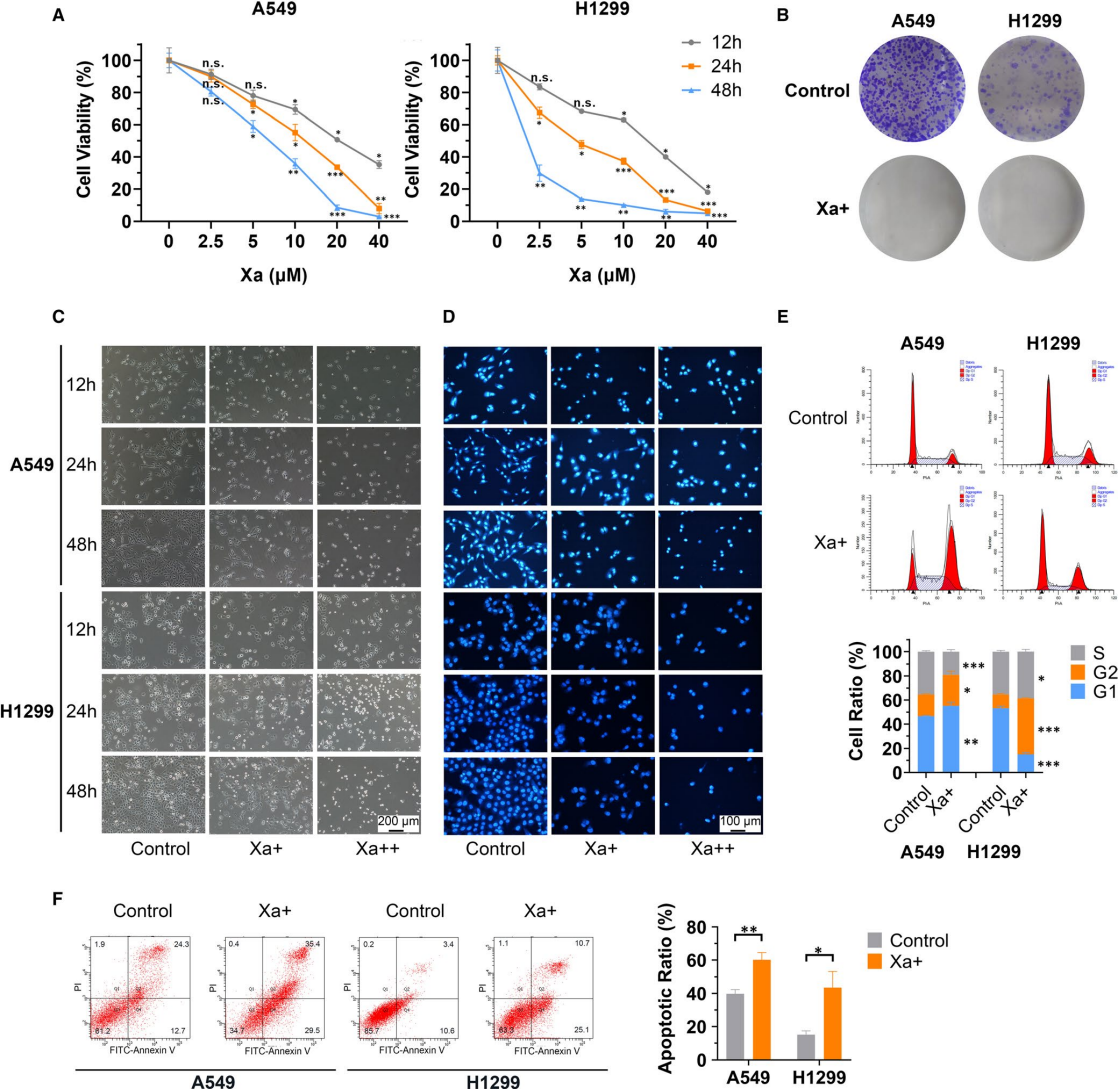
Xa suppresses cell proliferation and induces apoptosis in NSCLC cells. (A) CCK‐8 assay for evaluating the ability of Xa to inhibit the proliferation of A549 and H1299 cells at the indicated time points. (B) Colony formation assay showed Xa treatment could dramatically suppress the growth of A549 and H1299 cells. (C) Cell morphological changes induced by Xa were observed by an inverted microscope. (D) Hoechst 33258 staining of A549 and H1299 cells was performed to investigate the cell’s nuclear morphology after treatment with Xa. (E) Flow cytometry showed Xa treatment resulted in an obvious cell cycle block. (F) Flow cytometry showed Xa treatment resulted in an increased apoptosis rate. Experiments were repeated three times. *P < 0.05; **P < 0.01; ***P < 0.001

We firstly investigated the anti‐proliferative effect of Xa on NSCLC cell lines via CCK‐8 assays and found Xa dramatically suppressed cell growth in a dose‐ and time‐dependent manner (Figure 1A). The IC50 values were calculated for 12, 24 and 48 hours time points (Table S2). Based on IC50 of 24 hours, we determined the Xa concentration used for the following experiment: the low concentration (Xa+) means 1 × 24 h IC50 (10 μmol/L for A549 and 4 μmol/L for H1299), the high concentration (Xa++) means 2× 24 hours IC50 (20 μmol/L for A549 and 8 μmol/L for H1299). We subsequently performed colony formation assays and confirmed that Xa treatment suppresses the growth of A549 and H1299 cells (Figure 1B). Moreover, we found that Xa treatment remarkably changed cell morphology (Figure 1C). Likewise, we used Hoechst 33258 to identify the transitions of a cell’s nuclear morphology and found that Xa treatment led to an obvious increase of nuclear pyknosis (Figure 1D).

To further investigate whether Xa inhibits NSCLC cell proliferation by inducing cell cycle arrest or apoptosis, flow cytometry was performed to determine the cell cycle distribution and pro‐apoptotic effect of Xa in A549 and H1299 cells. The data showed that A549 cells were mainly blocked in G0/G1 phase while H1299 cells were mainly blocked in G2/M phase when treated with Xa, which may be attributed to the different sensitivity to Xa (Figure 1E). As shown in Figure 1F, a significant increase of apoptosis ratio was observed in both A549 and H1299 cells after treatment with Xa for 24 hours.

**FIGURE 2:**
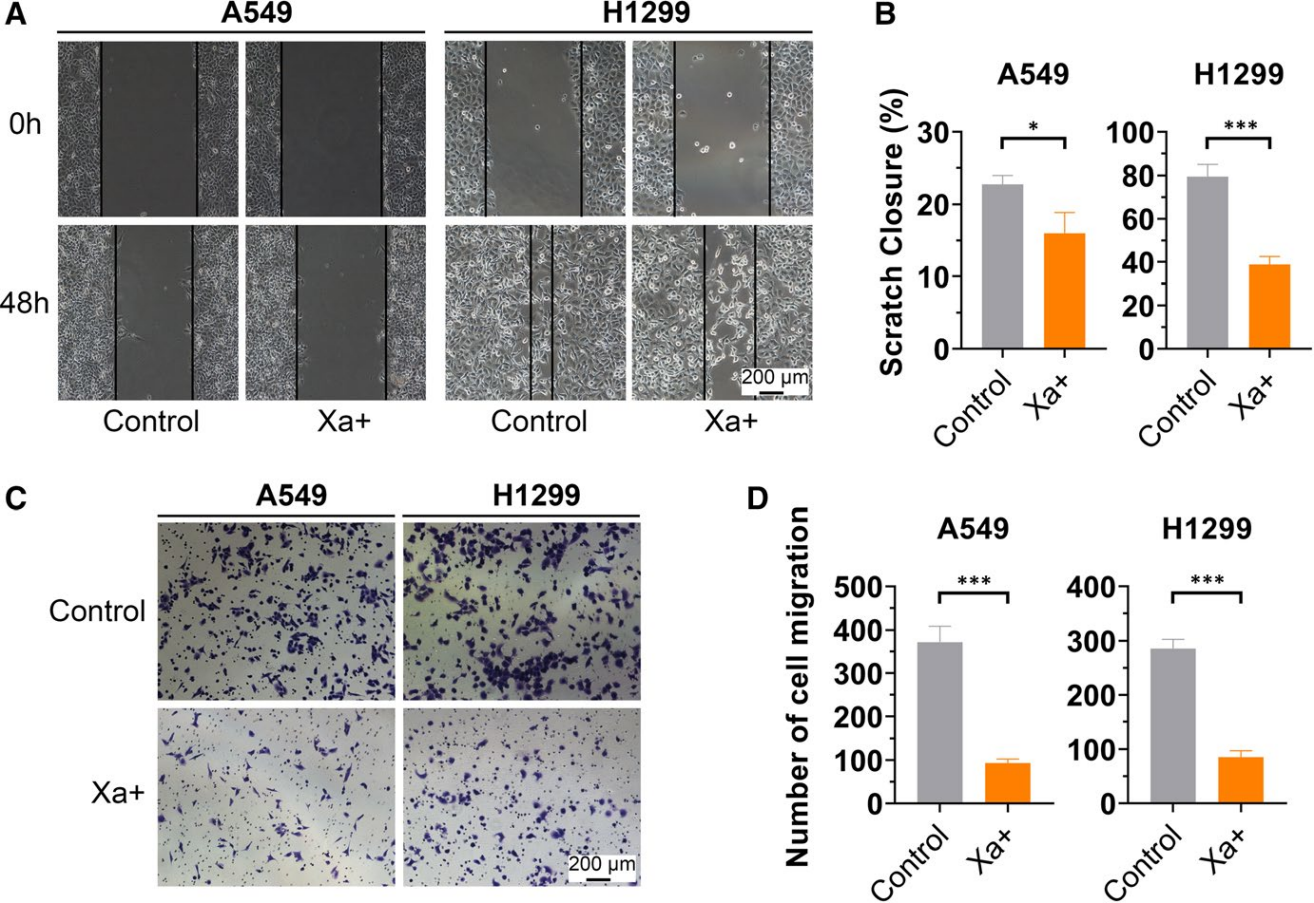
Xa suppresses cell migration and invasion in NSCLC cells. (A and B) Cell wound scratch assay showed the migration ability of A549 and H1299 cells were significantly decreased after treatment with Xa. (C and D) Transwell assay demonstrated Xa inhibited cell invasion of A549 and H1299. Experiments were repeated three times. *P < 0.05; **P < 0.01; ***P < 0.001

Additionally, we explored the effect of Xa on cell migration and invasion in NSCLC cells by using the wound scratch and transwell assays. Highly significant decreases in both migration (Figure 2A,B) and invasiveness (Figure 2C,D) were noted in Xa treated cells compared with control. Altogether, our in vitro findings suggested that Xa may act as an important antitumor agent for NSCLC.

### 3.2 Xa significantly activates p53 pathway and suppresses E2F targets in A549 cells

In order to explore molecular signature of NSCLC cells in responses to Xa, we constructed six cDNA libraries for RNA‐seq, with three replicates of Xa treatment and three replicates of controls in A549 cells. In total, 259.8 million 150 bp paired‐end clean reads were generated by RNA‐seq after removing low‐quality sequences (Table S3). On average, ~40% of the clean reads were mapped to the human genome (Table S3). After filtering with quantitated FPKM, 16 770 genes were considered to be expressed and selected for further analysis (Table S4). Among these genes, more than 88 % had FPKM values in the range of 1‐100 for each sample (Figure S1A).

**FIGURE 3:**
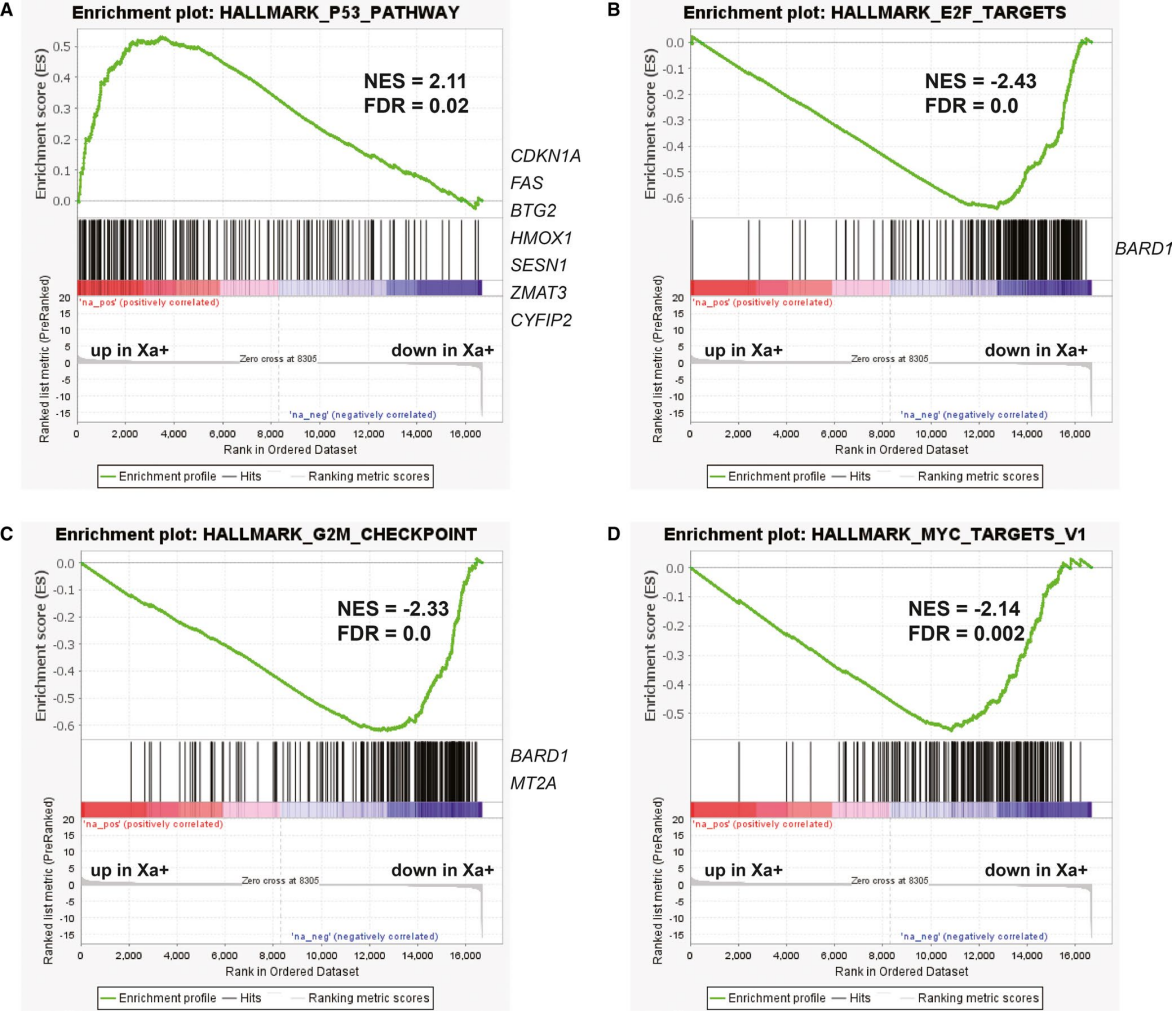
The GSEA plots indicate Xa significantly activates p53 pathway (A) and suppresses E2F targets (B), G2M checkpoint (C) and MYC targets (D) in A549 cells (FDR < 0.05). The positive normalized enrichment score (NES) indicates higher gene expression in the Xa treatment cells compared with control, and the negative NES was opposite. Contributing DEGs are shown

In total, we identified 78 up‐regulated and 55 down‐regulated DEGs in Xa treatment compared with control (Figure S1B; Table S5). GSEA indicated that the most up‐regulated genes are enriched in p53 pathway, which means that Xa can activate p53 pathway in A549 cells (Figure 3A). This is consistent with previous studies. Moreover, GSEA showed that Xa significantly suppresses E2F targets (Figure 3B), G2M checkpoint (Figure 3C) and MYC targets (Figure 3D). It’s worth noting that the down‐regulated DEG *BARD1* appears twice, which may be an uppermost contributor to this signature.

### 3.3 Xa inhibits BARD1 both at the mRNA and protein level

**FIGURE 4:**
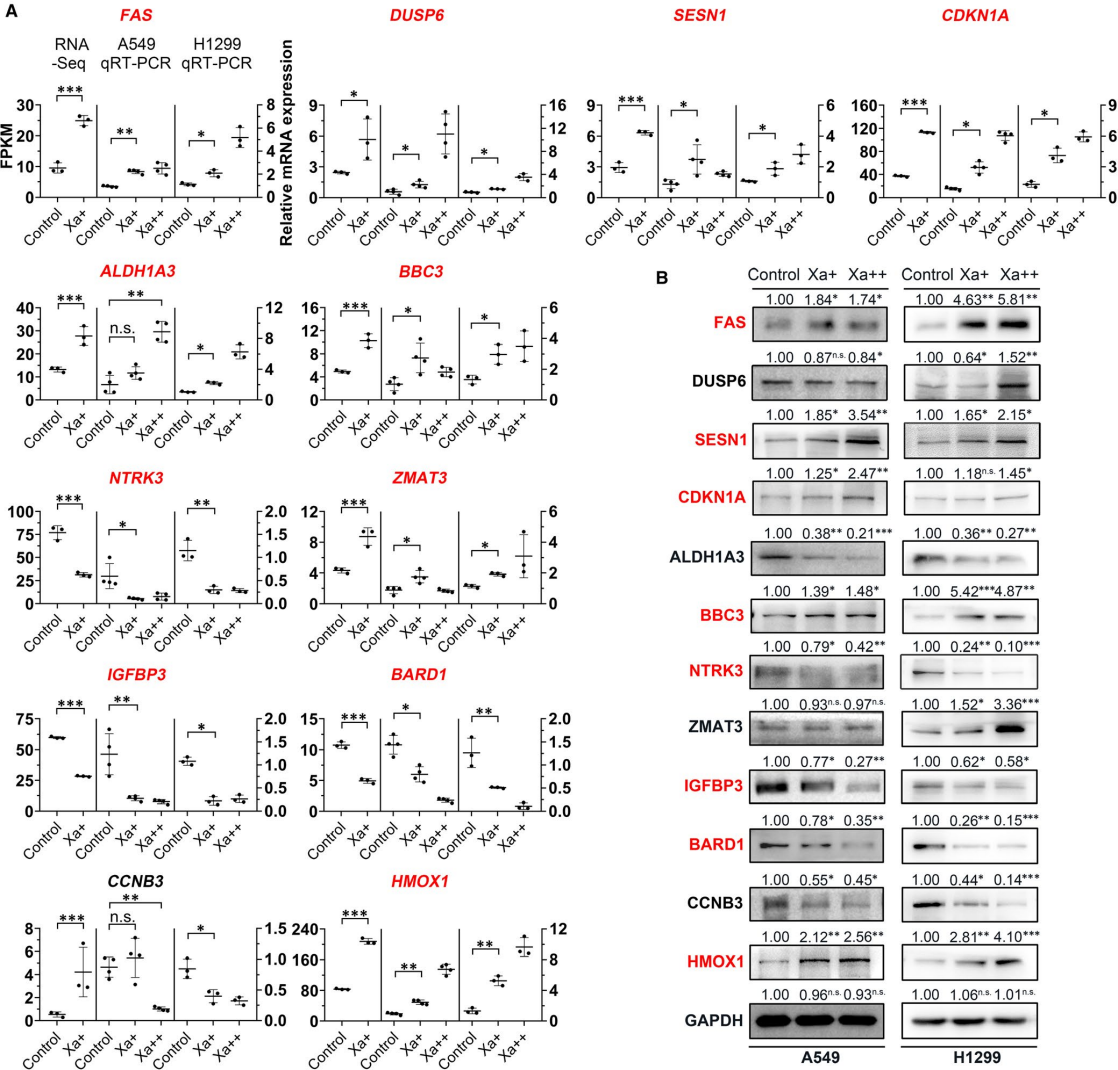
Validation of DEGs with qRT‐PCR and WB. (A) qRT‐PCR verification of 12 selected DEGs. FPKM of RNA‐seq is indicated on the *y*‐axis to the left. The relative qRT‐PCR expression level is shown on the *y*‐axis to the right. *GAPDH* was used as the internal control. (B) WB analysis of 12 selected DEGs. The genes highlight in red color indicated qRT‐PCR or WB were consistent with RNA‐seq results. *P < 0.05; **P < 0.01; ***P < 0.001

To validate the reliability of sequencing results and further explored the underlying molecular mechanisms, 12 DEGs (*NTRK3, HMOX1, ALDH1A3, DUSP6, BARD1, ZMAT3, FAS, IGFBP3, CCNB3, CDKN1A, BBC3, SESN1*) were selected for qRT‐PCR test. The detected expression patterns by qRT‐PCR and RNA‐seq were consistent for all genes except *CCNB3*, suggesting the reliability of the RNA‐seq results (Figure 4A). Next, we performed WB to make a further evaluation. WB results showed that the expression of 8 proteins including FAS, SSEN1, CDKN1A, BBC3, NTRK3, IGFBP3, HMOX1 and BARD1 were consistent with the RNA‐seq results (Figure 4B). Among these genes, BARD1 expression was inhibited both at the mRNA and protein level. Given the unique efficacy of BARD1 in HR pathway, we wonder whether BARD1 was involved in the antitumor effect of Xa on NSCLC cells.

### 3.4 Xa inhibits HR pathway and synergizes with Cis

**FIGURE 5:**
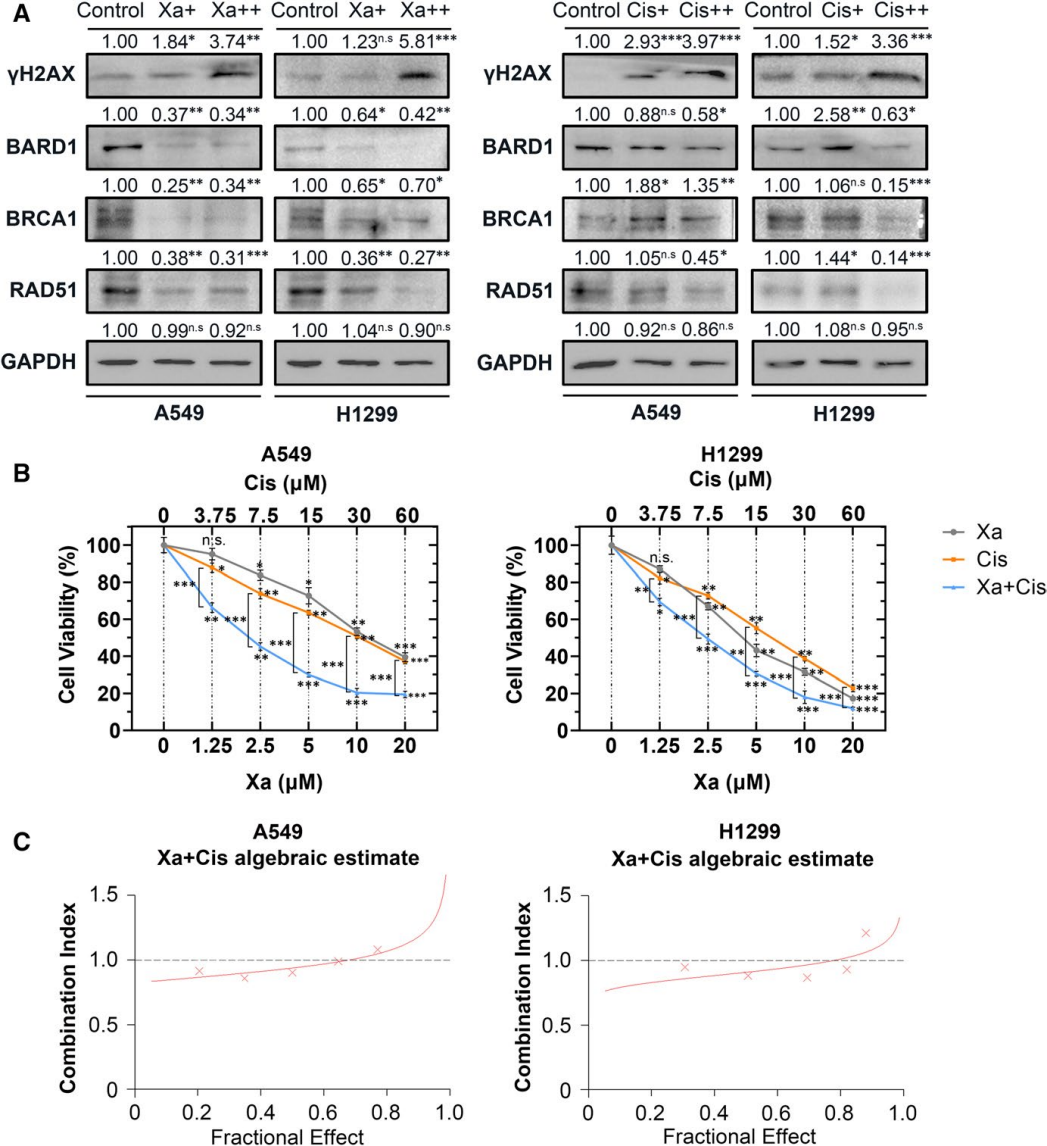
Xa inhibites HR pathway and synergizes with Cis. (A) WB analysis of γH2AX, BARD1, BRCA1 and RAD51, showing that both Xa (left panel) and Cis (right panel) caused DNA damage in a dose‐dependent manner. Xa inhibited HR pathway while Cis did not. (B) CCK‐8 assay, preliminary displaying the synergy between Xa and Cis. (C) Calcusyn was used to calculate the CI value of Xa and Cis, demonstrating that Xa and Cis exhibited a strong synergistic effect in NSCLC cells. Experiments were repeated four times. *P < 0.05; **P < 0.01; ***P < 0.001

We next examined the effect of Xa on the HR pathway. γH2AX was used as an indicator of DNA damage. WB results showed that both Xa and Cis cause DNA damage in a dose‐dependent manner (Figure 5A). Besides BARD1, we also found Xa can suppress the expression of BRCA1 and RAD51 at the protein level (Figure 5A), which indicates that Xa could inhibit HR pathway in NSCLC cells. However, cells treated with Cis did not possess significant effect on the expression level of these two proteins. (Figure 5A).

Considering the tight relationship between HR and Cis sensitivity, we hypothesized that Xa may increase NSCLC cells sensitivity to Cis by synergistic effect. We used Calcusyn to analyze the synergy between Xa and Cis. The results showed Xa and Cis exhibited a strong synergistic effect when they were applied to NSCLC cells at a concentration of 1:3 (Figure 5B,C). These results indicated that this antitumor effect might be related to the inhibition of BARD1 and HR pathway.

### 3.5 Xa inhibits HR pathway by down‐regulating BARD1 via JAK2‐STAT4 pathway

To evaluate the biological significance of BARD1 during Xa taking effect, we constructed a BARD1 overexpression (OE) pEZ‐M07 vector. Then we employed CCK‐8 assay to examine the effect of different drug combinations on NSCLC cell growth. Based on the CI value calculated by Calcusyn and the cell status after drug treatment, we determined the drug concentration used for studying drug synergism: 1.5 μmol/L Xa + 4.5 μmol/L Cis for A549, 1 μmol/L Xa + 3 μmol/L Cis for H1299.

**FIGURE 6:**
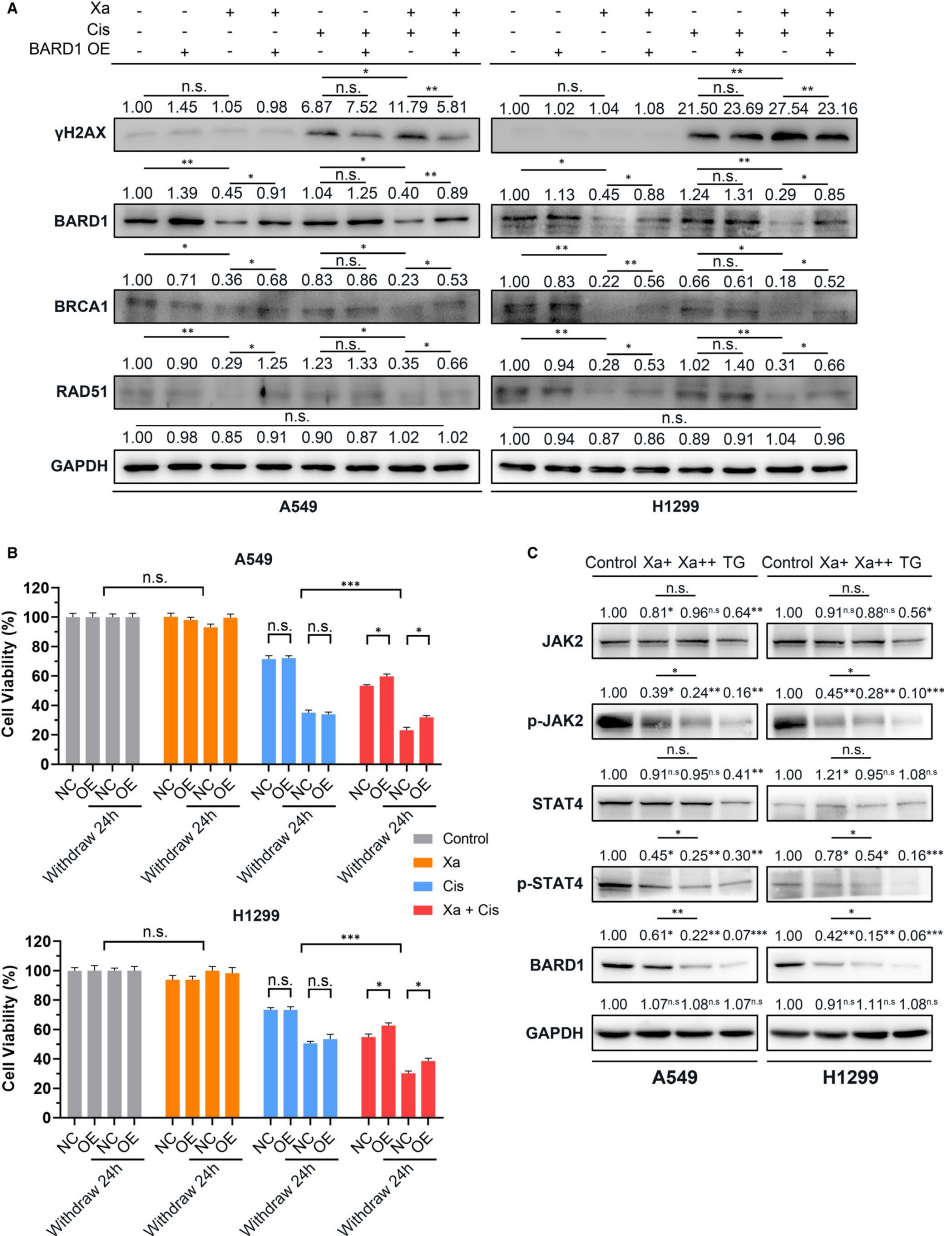
Xa inhibites HR pathway by down‐regulating BARD1 via JAK2‐STAT4 pathway. (A) WB analysis of γH2AX, BARD1, BRCA1 and RAD51, showing that BARD1 was successfully overexpressed, and overexpression of BARD1 (BARD1 OE) partially reversed the down‐regulation of BRCA1 and RAD51 caused by Xa. (B) CCK‐8 assay, displaying the effect of Xa was partially reversed by BARD1 OE. (C) WB analysis demonstrated that Xa reduces the protein level of phosphorylation of JAK2 (p‐JAK2) and p‐STAT4 and BARD1, which behaves similar to JAK2 inhibitor TG101348. Experiments were repeated four times. *P < 0.05; **P < 0.01; ***P < 0.001

WB results confirmed that BARD1 was successfully overexpressed (Figure 6A), and BARD1 OE partially reversed the down‐regulation of BRCA1 and RAD51 caused by Xa (Figure 6A). Moreover, CCK‐8 assays demonstrated that BARD1 OE weakened the effect of Xa + Cis, but had no significant effect on single Cis treatment (Figure 6B). Then, we performed an additional experiment through changing the fresh medium after 24 hours of drug treatment and testing after another 24 hours. The results preliminarily showed the suppressed state of DNA damage repair, which was almost accordance with the previous experiment (Figure 6B). It is worth noting that the extremely low concentration of Xa we used did not significantly cause cell proliferation inhibition and DNA damage, but already had the ability to increase Cis‐induced apoptosis (Figure 6B). These data indicated a unique and pivotal role of BARD1 during Xa taking its effect.

In order to verify the crucial function of HR, we knocked down RAD51 (si‐RAD51) on the basis of BARD1 OE to simulate a HR‐deficient status. WB results showed that RAD51 was successfully knocked down (Figure S2A). CCK‐8 assays demonstrated that silencing RAD51 reversed the effects of BARD1 OE (Figure S2B). These findings confirmed that BARD1 exerts its effects through the HR pathway.

We next focused on how Xa inhibits the expression of BARD1. We used PROMO (alggen.lsi.upc.es) to identify STAT4 as a potential transcription factor for BARD1. Further experiment showed that after Xa treatment, expression of JAK2 and STAT4 did not change, but the phosphorylation of JAK2 and STAT4 was significantly reduced (Figure 6C), which behaves similar to JAK2 inhibitor TG101348 (Figure 6C). These data demonstrated that Xa may repress the expression of BARD1 via JAK2‐STAT4 pathway.

## 4 DISCUSSION

Xa is a sesquiterpene lactone compound and it can work as an alkylating agent because of its alpha‐methylene‐gamma‐butyrolactone moiety (αMγL) structure. This structural basis makes Xa possess a great potential for inducing DNA damage.^26^ Previous investigations demonstrated that Xa exerts its anticancer effect by inducing DNA damage, reticulum stress, oxidative stress and so on.^19^, ^20^, ^21^, ^27^ However, the effects of Xa on the transcriptome of cancer cells have not been reported. In this study, we aimed to elucidate the antitumor effects of Xa and its underlying mechanisms in NSCLC cells. Firstly, we found that Xa inhibits cell proliferation, migration, invasion and progression of the cell cycle in NSCLC cells. Subsequently, we used RNA‐seq to assess the changes of gene expression profiles induced by Xa treatment in NSCLC cells. We identified 133 genes to be differentially expressed (78 up‐regulated and 55 down‐regulated in Xa treatment). And these DEGs were mainly involved in DNA damage response, cell apoptosis and p53 signaling pathway. Moreover, GSEA showing that Xa significantly activates p53 pathway and suppresses E2F targets, G2M checkpoint and MYC targets in A549 cells. It has been reported that Xa induces apoptosis via p53 signaling pathway, which was consistent with our results.^28^ Therefore, we focused on exploring the underlying molecular mechanism of Xa’s antitumor effects. For this purpose, 12 DEGs which may be the potential targets of Xa were selected for qRT‐PCR and WB to verify the reliability of RNA‐seq. Among these genes, the down‐regulated gene *BARD1* seems to be particularly interesting in light of our results and its vital role in HR pathway. The relationship between Xa and HR pathway has not yet been reported. Thus, we chose it for further study.

Recent studies have revealed the important links between BARD1 and HR pathway. BARD1 and BRCA1 are known to function in HR by forming a stable heterodimer through their RING‐finger domains then co‐localize at nuclear foci.^11^, ^29^ The role of BRCA1‐BARD1 during HR is to produce a single‐stranded template by facilitating the nucleolytic resection of DNA ends, and then recruit other important tumor suppressor complex, including BRCA2‐PALB2 and RAD51.^30^, ^31^, ^32^ BRCA1 requires BARD1 to function, and BRCA1 is unstable and rapidly degrades in the absence of BARD1.^31^ Meanwhile, BRCA1‐BARD1 can interact with RAD51 and enhances its activity.^11^ RAD51 executes an essential task in HR, interacts with other HR‐related proteins and captures the backup DNA copy, which matching the sequence of the broken strand with a homologous sequence in the intact DNA double helix.^11^ Considering the central role of RAD51, it is universal and reasonable that RAD51 can affect most proteins which are active in HR.^33^, ^34^, ^35^, ^36^ Strikingly, RAD51 is overexpressed in most cancer cells including NSCLC, and its overexpression generally leads to the genomic instability and resistance to DSB‐inducing therapies.^37^ In the present study, we highlighted that Xa could suppress BARD1 expression, and subsequently weaken the expression of BRCA1 and RAD51, indicating the involvement of HR pathway in Xa’s antitumor effects.

It is known that DNA damage‐inducing agents Cis had no significant effect on the HR pathway. Meanwhile, the inhibition of HR pathway in NSCLC cells is associated with the increased cell sensitivity to Cis. In fact, there are historical precedents for the successful treatment of cancer by inhibiting DNA repair pathway. For instance, poly ADP‐ribose polymerase (PARP) inhibitors are effective drugs against breast cancer patients who have lost HR capability due to the mutations of BRCA1/2 but simultaneously with enhanced activity of PARP.^38^, ^39^ Therefore, we explored the synergistic effect between Cis and Xa and found that Xa synergized with Cis when the ratio of Xa/Cis kept 1:3. And this synergistic effect could be partly reversed after BARD1 OE (Figure S2A,B), which further confirmed our speculation. Taken together, these results suggest that Xa can inhibit HR via BARD1/BRCA1/RAD51 axis and enhance NSCLC cell sensitivity to Cis. This finding may reduce cytotoxic effects and improve clinical outcomes of Cis in the future.

**FIGURE 7:**
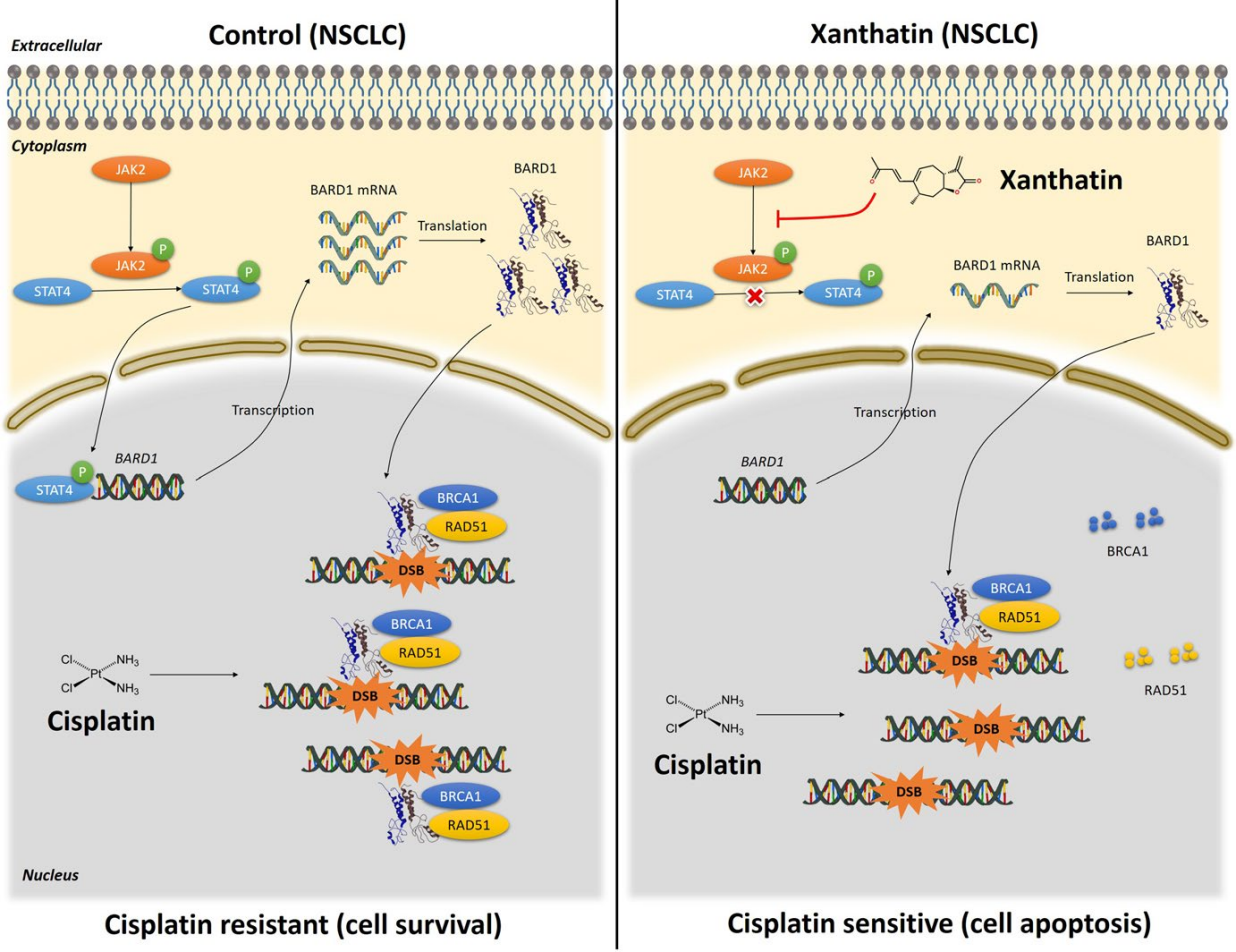
A schematic model of the molecular mechanism underlying the role of Xa in NSCLC cells. Left panel shows drug resistant to Cis of NSCLC due to the abnormal enhancement of DNA repair capacity in cancer cells, leading to cell survival. Right panel shows Xa suppresses HR and synergizes with Cis. Mechanistically, Xa inhibites the conversion of JAK2 to p‐JAK2, making STAT4 unable to form its active form p‐STAT4. Then, the expression of BARD1 is decreased due to the lack of the transcription factor p‐STAT4, leading to reduction of the BARD1 protein. As a result, assembling of BARD1‐BRCA1 complexes and the recruitment of RAD51 at double‐strand breaks (DSBs) sites were decreased, resulted in degradation of BRCA1 and RAD51. Eventually, the down‐regulation of BARD1 triggered by Xa decreases the ability of cells against DSBs and increases the NSCLC cells sensitivity to Cis, leading to cell apoptosis

The Janus kinase (JAK)‐signal transducer and activator of transcription (STAT) signaling pathway plays a vital role in many biological processes, including regulating angiogenesis, modulating inflammatory, immune responses, hematopoiesis and even oncogenesis. Recent studies have shown that STATs can function as transcription factors after phosphorylating, dimerizing and nucleus translocating.^40^, ^41^ Importantly, STAT4 is predicted as a transcription factor for BARD1 in NSCLC cells by using the PROMO, and there is evidence that the expression of STAT4 was persistently activated in NSCLC cells through its co‐activator JAK2.^42^, ^43^ Interestingly, it has been reported that Xa is a covalent and selective inhibitor of JAKs.^44^ Thus, to further gain insights into the mechanism of Xa‐mediated inhibition of BARD1, we characterized the expressions of key proteins in JAK2‐STAT4 pathway. Our investigation demonstrated that disruption of JAK2‐STAT4 pathway by Xa contributed to its inhibitory effects on the expression of BARD1 and HR pathway in NSCLC cells (Figure 7).

In addition, it is worth noting that Xa can inhibit DNA damage repair as a DNA damage inducer, which might account for the different cell cycle arrests under the treatment of Xa in NSCLC cells. Actually, similar results had also been previously reported.^20^ Mechanistically, overproduction of DSBs can cause a strong cell cycle arrest at the G0/G1 phase,^45^ while HR‐deficient cells are easily blocked at the G2/M phase because HR occurs between sister chromatids.^12^ In the present studies, we speculated that high concentration of Xa potently induced cell cycle arrest at G0/G1 checkpoint in A549 cells via producing DSBs, which covered up the effect of HR deficient. And low concentration of Xa induced cell cycle arrest at G2/M checkpoint in H1299 cells via inhibiting HR pathway, because the IC50 value of H1299 is much lower than A549. These findings suggested that DNA damage induced by Xa should not be ignored. In fact, RNA‐seq and validation results indicated that DNA damage caused by Xa leads to a series of gene expression changes through p53 pathway. Numerous p53 target genes were up‐regulated by Xa treatment, including pro‐apoptotic genes ZMAT3, FAS,^46^ BBC3,^47^ cell cycle regulatory genes CCNB3,^48^ CDKN1A^49^ and anti‐stress gene SESN1.^50^ However, similar cell proliferation inhibition were also observed in the p53‐deficient H1299 cells, the effect of p53 on these changes remains to be determined. It had been reported that p53‐deficient tumor cells rely much more on ATR/Chk1 to arrest cell progression.^19^ Therefore, More experiments are needed to further elucidate the influence of Xa on gene expression.

In summary, here we first proposed the potential of Xa as a sensitizer for Cis because of its significant ability to inhibit HR. Subsequently, our findings indicated that Xa inhibits HR via JAK2/STAT4/BARD1 axis and enhances cell sensitivity to Cis (Figure 7). These data help further reveal the antitumor effect and mechanism of the sesquiterpene lactone compound Xa on NSCLC cells, which provides insight for Xa as a potential anticancer agent for NSCLC, especially in combination with routine chemotherapy. However, further investigations especially in vivo evidence are needed.

## CONFLICT OF INTEREST

The authors declare that there is no conflict of interests.

## AUTHOR CONTRIBUTIONS

**Jian Zhang:** Formal analysis (equal); Investigation (equal); Methodology (equal); Project administration (equal); Software (equal); Supervision (equal); Visualization (equal); Writing‐original draft (equal); Writing‐review & editing (equal). **Sheng Yang:** Data curation (equal); Formal analysis (equal); Investigation (equal); Methodology (equal); Validation (equal); Visualization (equal); Writing‐original draft (equal); Writing‐review & editing (equal). **Hongmei Guan:** Investigation (equal); Methodology (equal); Validation (equal); Writing‐review & editing (equal). **Jue‐Yu Zhou:** Investigation (equal); Methodology (equal); Supervision (equal); Writing‐review & editing (equal). **Yuan Gao:** Conceptualization (equal); Data curation (equal); Funding acquisition (equal); Investigation (equal); Methodology (equal); Project administration (equal); Supervision (equal); Writing‐original draft (equal); Writing‐review & editing (equal).

## Supporting information

Fig S1‐S2

Table S1‐S5

## Data Availability

The data that supports the findings of this study are available in the supplementary material of this article.
