# Supplementary material for: Xanthatin synergizes with cisplatin to suppress homologous recombination through JAK2/STAT4/BARD1 axis in human NSCLC cells
Source: J Cell Mol Med. 2021 Jan 13;25(3):1688–99. doi: 10.1111/jcmm.16271 (PMC7875932; doi:10.1111/jcmm.16271)
Supplement: Supplementary file 1 — Fig S1‐S2 [file JCMM-25-1688-s001.docx]

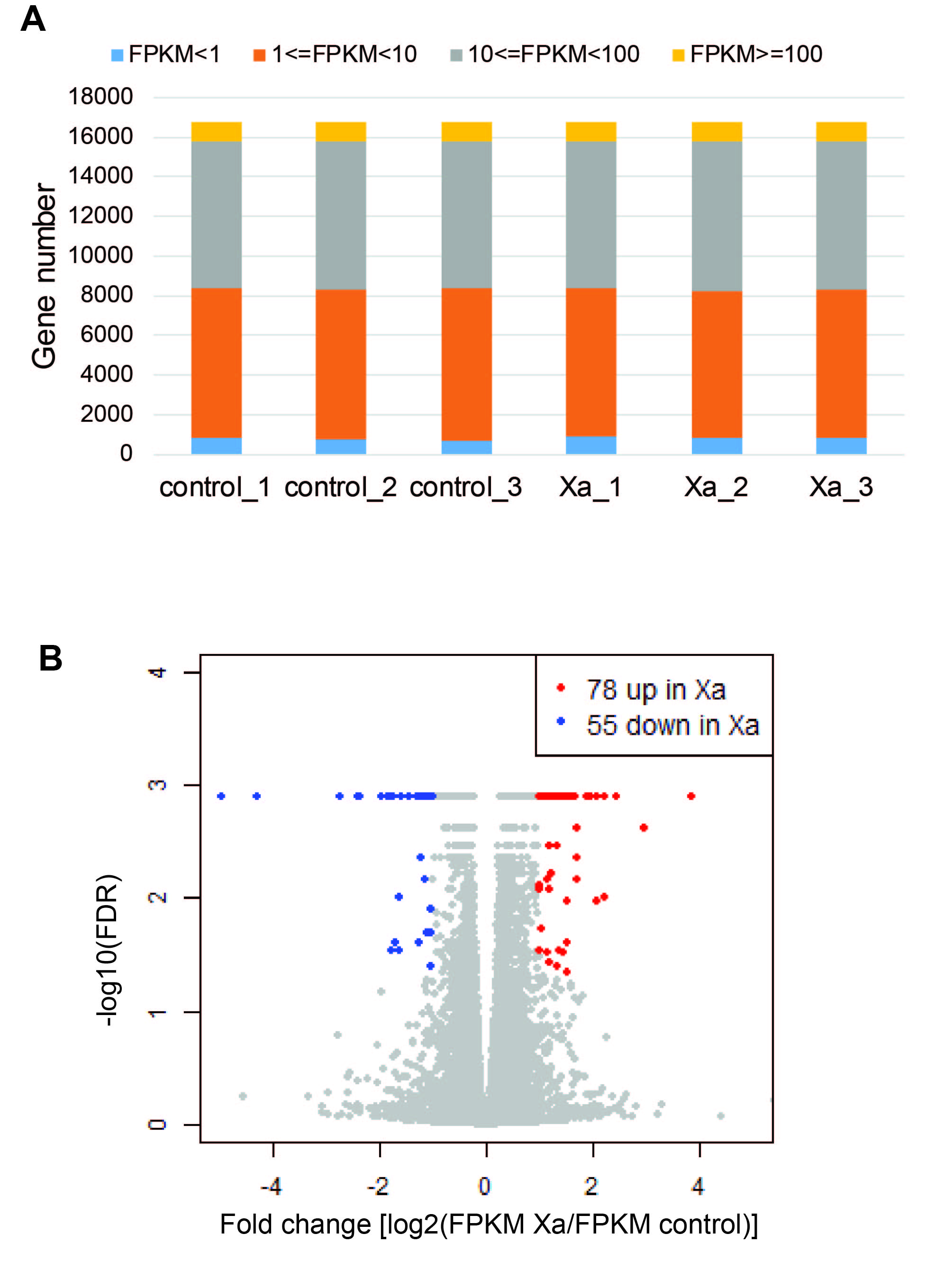


Figure S1. RNA-Seq analysis of Xa treatment and control in A549 cells. (A) Number of expressed genes in each sample. (B) 78 up-regulated and 55 down-regulated differentially expressed genes were identified in Xa treatment cells compared with control.


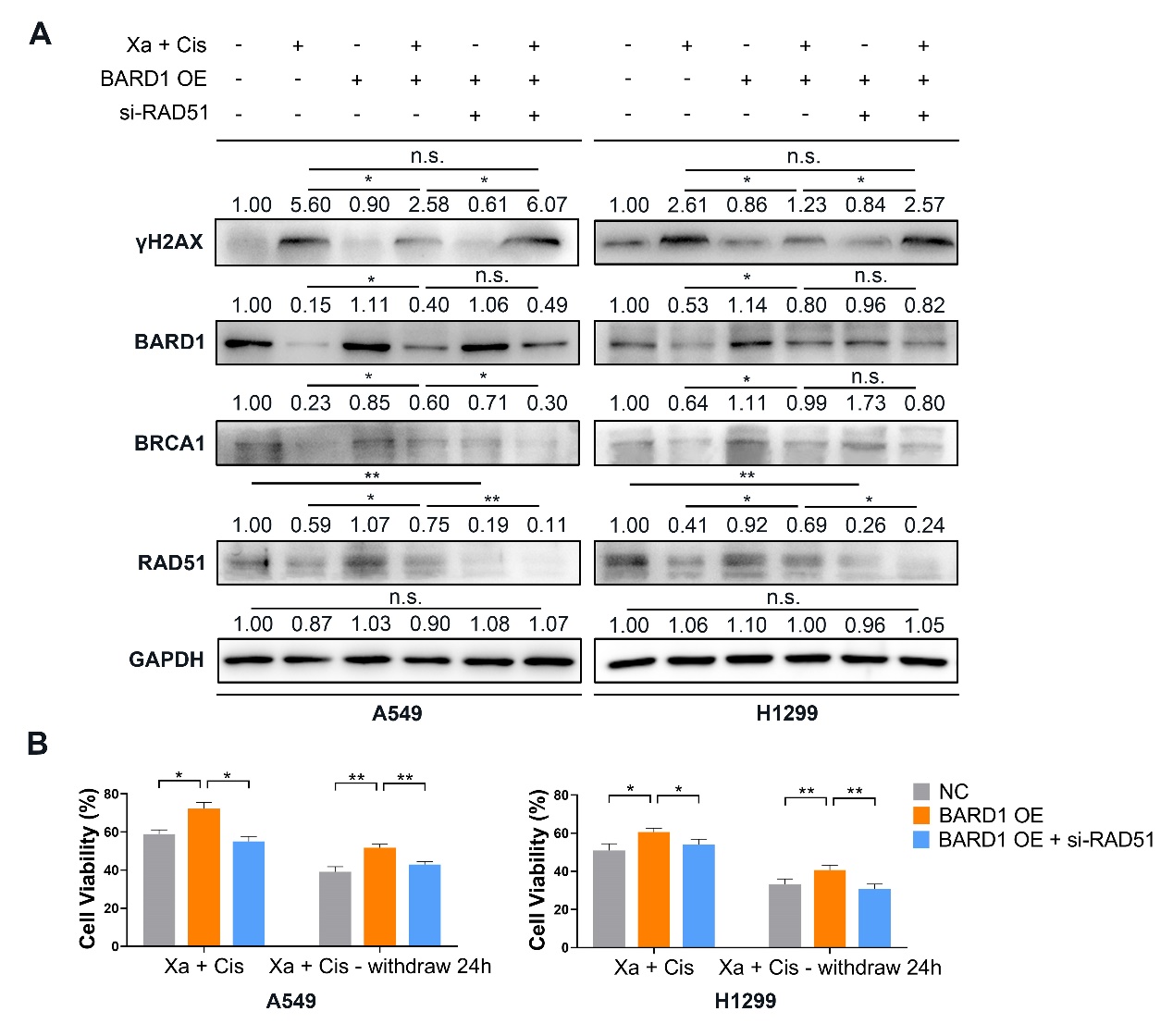


Figure S2. Knocking down RAD51 (si-RAD51) almost eliminated the reversal effect of BARD1 overexpression (OE). (A) WB analysis of γH2AX, BARD1, BRCA1 and RAD51 under different combinations of Xa + Cis, si-RAD51 and BARD1 OE treatment. (B) [CCK-8 assay](https://www.sciencedirect.com/topics/neuroscience/mtt-assay) for evaluating the ability of cell proliferation under different combinations of Xa + Cis, si-RAD51 and BARD1 OE treatment.
